# Nitrogen deposition does not alleviate the adverse effects of shade on *Camellia japonica* (Naidong) seedlings

**DOI:** 10.1371/journal.pone.0201896

**Published:** 2018-08-09

**Authors:** Cuiju Liu, Xiao Guo, Kuiling Wang, Yingkun Sun, Wei Li, Qingchao Liu, Qinghua Liu

**Affiliations:** College of Landscape Architecture and Forestry, Qingdao Agricultural University, Qingdao, China; Shandong University, CHINA

## Abstract

*Camellia japonica* (Naidong), a Tertiary relict species with a unique biological and cultural characteristic, is a special ecotype of *C*. *japonica* and is the northernmost distributed populations of *C*. *japonica* in the world. This study investigated the interactive responses of *C*. *japonica* (Naidong) to shade and nitrogen deposition focusing on seedling growth, leaf morphology and leaf physiology under two light regimes (15% and 65% of full sunlight to represent deep shade and slight shade respectively) and three nitrogen deposition regimes (0, 6 and 12 g N m^-2^ year^-1^) in a greenhouse. After 123 d of treatment, the results showed that the deep shade reduced the growth of seedlings significantly compared to slight shade, but improved the specific leaf area, leaf water content, chlorophyll content and F_v_/F_m_ of plants. Moderate nitrogen (6 g N m^-2^ year^-1^) supply increased the crown area, specific leaf area, leaf water content, chlorophyll content and water use efficiency of seedlings. However, high nitrogen (12 g N m^-2^ year^-1^) supply reduced the basal diameter, crown area, specific leaf area and leaf water content. No significant interaction of shade and nitrogen deposition on *C*. *japonica* (Naidong) was found. There is a threshold of nitrogen deposition for the growth of *C*. *japonica* (Naidong). *Camellia japonica* (Naidong) populations should be protected by collecting of germplasm resources and carrying out the *ex situ* conservation.

## Introduction

Emissions and deposition rates of nitrogen are predicted to double from current values by 2050 due to the anthropogenic sources, for examples, combustion of fossil fuels, production and consumption of chemical nitrogen fertilizers, and rapid development of agriculture [1–5]. The amount of nitrogen deposition in the worldwide differs evidently among different regions, and nitrogen deposition in eastern Asia is increasing remarkably [4, 6–8]. China is experiencing intense air pollution caused in large part by anthropogenic emissions of reactive nitrogen [3, 5, 6, 9]. In recently years, nitrogen deposition in the eastern coastal areas of China, particularly in offshore areas, has become more severe [5, 8, 10]. The report indicated that the total nitrogen deposition flux was 204.51 mg m^-2^ mon^-1^ in the Yellow Sea of China during 2001–2012 [10].

Nitrogen supply affects plant reproduction, survival and biodiversity, which leads to both advantages and disadvantages. On one hand, elevated nitrogen deposition may stimulate seed germination, and facilitate plant growth and productivity [1, 11–14]. On the other hand, excess nitrogen may cause many environmental problems, such as plant nutritional imbalance, plant growth depression, decline in biodiversity, and ecosystem function disorder [2, 3, 11, 12, 14].

Plant species growing in natural habitats often distributes in different light environments (e.g. open forest, forest gap, forest edge, under forest). Light heterogeneity in a forest increases as a result of natural disasters and human activities, and heterogeneity in the light environment might be important for expression of some plant traits [15]. Variation in irradiance has a fundamental effect on development, production and survival of plant, and excessively low or high incident light will prevent photosynthesis in the plant leaves [14, 16, 17]. A given species usually exhibits a variety of morphological and physiological characteristics under changeable light conditions.

Studies with respect to the effects of the interaction of nitrogen deposition and shading on plant physiological parameters have been reported [14, 18–20]. Previous studies indicated that the efficiency of utilization nutrients is dissimilar in different light environments [18, 19]. Thus, diverse mechanisms by which plants respond to variation in light and nitrogen deposition resources. One study indicated that nitrogen addition can alleviate the negative effects of deep shading on lettuce, because the nitrogen supply enhanced the photosynthetic rate, leaf area and nitrate content of *Lactuca sativa* L. Var. youmaicai [18]. Nitrogen addition is reported to increase the height and whole dry mass of five tropical dry forest tree species under low irradiance because the plants were more limited by nitrogen supply rather than incident light [19]. While another study found that the increased light availability had a positive effect on both the growth rate and final biomass of *Deschampsia flexuosa*, regardless of nitrogen treatment [21]. Ma *et al*. researched the light and nitrogen availability influence leaf biochemistry and the efficiency of the CO_2_ concentrating mechanism in *Miscanthus*×*giganteus* [22].

*Camellia japonica* is mainly distributed in eastern coastal areas of China, two Japanese islands (Shikoku and Honshu), and southern Korean peninsula [23, 24]. *Camellia japonica* (Naidong) (hereafter called Naidong) is an evergreen broad-leaved shrub or dungarunga with unique biological and cultural characteristic [23, 25]. As a Tertiary relict species, Naidong is a special ecotype of *C*. *japonica* and represents the northernmost distributed populations of *C*. *japonica* in the world. Naidong were more tolerant to low temperature than other *C*. *japonica*, which have a long flowering period and blooms in winter. Compared with other natural *C*. *japonica* populations, Naidong has higher genetic diversity [26]. The distribution of Naidong is limited to several islands of the Yellow Sea of China because of the distinctive climatic characteristics of the territory [25]. However, a great number of its natural populations have disappeared mainly due to habitat fragmentation or destruction of the natural environment, resulting in only 506 extant plants of Naidong on the islands of Qingdao, China [27]. Naidong grows in a highly heterogeneity light environment because of the fragmentation of habitats [27–30]. Due to the low number of Naidong individuals, its populations are at great risk of extinction given the challenges of increasing of light heterogeneity in habitats and accelerating of nitrogen deposition in the offshore area of China.

To our knowledge, no studies have been made on the morphological and physiological responses of Naidong seedlings to various combinations of shade and nitrogen deposition conditions. Thus, two-year old Naidong seedlings were subjected to different light intensities and nitrogen addition rates, and growth parameters, leaf morphological traits, chlorophyll content, photosynthetic characteristics, and chlorophyll fluorescence parameters were measured. The aims of this study were to determine 1) how Naidong seedlings acclimate to different light or nitrogen deposition conditions; 2) whether nitrogen load alleviates the effects of shade on Naidong; and 3) the implications for the protection of the precious remaining natural populations of Naidong.

## Materials and methods

### Study site and plant materials

We state clearly that no specific permissions were required and the field studies did not involve any endangered or protected species. Moreover, we had no vertebrate studies in this research.

The study was conducted at Qingdao Agricultural University (36°31′N, 120°39′E), Qingdao city, Shandong, China. The site experiences a warm temperate monsoon climate, with an average temperature of 12.7 °C, and an average annual precipitation of 700 ± 100 mm, most of which falls during the summer [27, 31]. The experiment was carried out in the greenhouse at the experimental station to maintain a controlled environment. The greenhouse was well ventilated by rolling up the plastic films on the sides.

On September 2013, Naidong seeds were collected from Changmenyan Island (36°10′N, 120°56′E), Qingdao city, Shandong, China. The seeds were stored at 0–4°C over winter. The seeds were disinfected with 1% potassium permanganate solution and soaked in distilled water for 5–7 d in December 2013. The seeds were stimulated to germinate by stored in wet sand. In March 2014, the germinated seedlings were transferred into plastic pots (150 mm height × 150 mm diameter) with one seedling per pot. Each pot was filled with a mixture of 1:1 (v/v) raw soils and peat. Then, the Naidong seedlings grow in the greenhouse with regular maintenance. Two-year seedlings that were healthy and uniform in growth were selected for use in present study in 2016. Throughout the experiments period, all pots received adequate watering. Weeds and insects were controlled manually.

### Experimental design

A factorial experiment was conducted incorporating two factors (light and nitrogen addition) in a 2×3 completely randomized design with nine replications per treatment, with a total of 54 pots. Two light levels (65 and 15% of full sunlight; L1 and L2, respectively) and three nitrogen deposition rates (0, 6 and 12 g N m^-2^ year^-1^; N1, N2 and N3, respectively) were applied. The two light levels represented slight shade (L1) and deep shade (L2), respectively. L1 and L2 conditions were simulated with the different density of woven black nylon net shelter placed over the seedlings in the greenhouse, and the light intensity was measured with a Quantum/Foot-Candle Meter (Spectrum Technologies, Inc. USA). For each light level, we applied the three nitrogen treatments: N1 represented the control; N2 was representative of nitrogen deposition rates already recorded in some areas of China; and N3 represented a high deposition rate that may be attained in the future [3, 5, 12, 32].

According to the study, Naidong seedlings grow and develop mainly from June to September [33]. Therefore, the experiment was carried out from 5 June to 5 October. Beginning on 5 June, the nitrogen treatments were applied every half month and eight times in total, ending on 5 October. According to previous reported, the ratio of ammonium nitrogen (NH_4_-N) to nitrate nitrogen (NO_3_-N) in atmospheric nitrogen deposition was about two in China recent years [5, 12]. Nitrogen deposition was simulated by adding mixed solutions of (NH4)_2_SO_4_ and KNO_3_ (1:1, M/M). In addition, K_2_SO_4_ and KCl solutions of different concentrations were also added to different nitrogen treatments to ensure that all treatments received the same amount of potassium as well as sulfur. Solutions corresponding to 1/24 of the annual nitrogen deposition were added at each application. The compositions and concentrations of the solutions applied in the three nitrogen deposition treatments during the experiment are shown in Table 1.

**Table 1:** Compositions and concentrations of the solutions applied to the four nitrogen treatments during the experiment.

Nitrogen treatment (g m^-2^ year^-1^)	Solution composition	Concentration (mol L^-1^)
0	K_2_SO_4_	2.47×10^−3^
6	(NH_4)2_SO_4_	8.18×10^−4^
	KNO_3_	8.18×10^−4^
	K_2_SO_4_	1.64×10^−3^
	KCl	8.18×10^−4^
12	(NH_4_)_2_SO_4_	2.47×10^−3^
	KNO_3_	2.47×10^−3^
	KCl	2.47×10^−3^

### Measurements

Seedlings height (H, from ground level to the apical bud), basal diameter (BD, about 1 cm above the ground level) and crown area (CA) were measured separately approximately every 20 days in all experimental treatments. The CA was calculated as: crown area = 0.25 π×a×b where a and b are the length of the diagonals [11]. Throughout the experimental period, plant growth parameters were recorded at six times points, and eight or nine seedlings were measured for each treatment.

Leaf morphology traits were measured in the mid-August. Seven to nine fully expanded leaves (the third or/and fourth leaves from the tip) per treatment were sampled with a Yaxin-1241 portable leaf area meter (Yaxin Inc., Beijing, China). The leaf fresh weight (LFW) was measured with an electronic balance. The leaves were first dried at 105°C for 0.5 h for deactivation of enzymes, and then oven-dried at 80 °C for 24 h to calculate the leaf dry weight (LDW). Subsequently, the specific leaf area (SLA = LA / LDW) and leaf water content (LWC = (LFW—LDW)/LFW) were calculated.

Seven to nine fully expanded leaves (the second or third leaf from the shoot tip) per treatment were selected for measurement of gas exchange parameters, and were measured with a CIRAS-3 portable photosynthesis system (PP Systems, Amesbury, MA, USA) between 8:30 and 11:30 am on cloudless days in early August. Gas-exchange characteristics, including assimilation rate (*A*), transpiration rate (*E*), stomatal conductance (*g*_s_), interal CO_2_ (*C*_*i*_) and water use efficiency (WUE) were automatically recorded by instrument. Light was supplied from fully automatic red/blue/green/white LED light source at the irradiance (photosynthetically active radiation) of 1000 μmol·m^−2^·s^−1^. All data were measured at average temperature inside the chamber of 27 °C, relative humidity of 60% and CO_2_ concentration inside the chamber of ambient level (approximate 400 μmol·mol^-1^).

Chlorophyll fluorescence parameters were determined using a Pocket PEA (Hansatech Instruments Ltd, King’s Lynn, UK). Leaves were kept in the dark for 30 min to ensure complete relaxation of all reaction centers before measurements on sunny days [34]. The initial fluorescence (*F*_0_), variable fluorescence (*F*_v_), maximal fluorescence (*F*_m_) and maximum quantum yield of photosystem II (*F*_v_/*F*_m_) were determined. The maximum quantum yield of photosystem II (PSII) was calculated as (*F*_m_—*F*_0_)/*F*_m_, which reflects the intrinsic PSII efficiency [6].

Seven to nine fully expanded leaves (the second or third leaf from the shoot tip) in each treatment were sampled for determination of leaf chlorophyll content, and three fully expanded leaves were sampled for determination of leaf nitrogen concentration (LN) and leaf phosphorus concentration (LP). The photosynthetic pigment was extracted in late August using the ethanol extraction method [12], and quantified using a UH5300 UV/VIS spectrophotometer (Hitachi, Inc., Tokyo, Japan). The LN and LP concentrations were measured using the Kjeldahl method and the molybdenum antimony-D-isoascorbic acid colorimetry method, respectively [12].

### Statistical analysis

Two-way analysis of variance (ANOVA) was applied to evaluate the effects of light intensity, nitrogen deposition and their interaction. One-way ANOVA and Duncan’s multiple range tests were conducted to analyze the differences among the six treatments, performed at a level of significance of 0.05. Before ANOVA, data were checked for normality and homogeneity of variance. When necessary, log transformation or square root transformation was applied. All of the statistical analyses were performed using the IBM SPSS Statistics 21.0 software package (IBM Corporation, Armonk, NY, USA). All figures were drawn with the Origin 9.0 software (OriginLab Co., Northampton, MA, USA).

## Results

### Plant growth

The basal diameter and crown area of Naidong seedlings showed significant responses to light intensity and nitrogen deposition (Table 2). However, no significant effect of light intensity, nitrogen addition, and the interaction was observed on seedling height. The seedlings basal diameter and crown area under high irradiance were greater than those observed under low irradiance. The nitrogen addition mainly affected the crown area, as the crown area in the control and moderate nitrogen addition groups was significantly higher than that of other groups under slight shade (Table 3).

**Table 2:** Two-way ANOVA of the effects of light intensity, nitrogen deposition rate and their interaction on growth and physiological characteristics in *C*. *japonica* (Naidong) seedlings.

Parameters	*F* and it’s significance		
	N	L	L×N
Height (cm)	0941^ns^	0.002^ns^	0.451^ns^
Basal diameter (cm)	5.799*	9.852*	0.507^ns^
Crown area(cm^2^)	3.277*	18.122**	1.646^ns^
SLA (mm^2^ g^-1^)	0.647^ns^	10.910*	2.123^ns^
LWC	1.715^ns^	29.091**	4.150*
*A* (μmol m^-2^s^-1^)	1.137^ns^	0.496^ns^	0.929^ns^
*E* (mmol m^-2^s^-1^)	1.738^ns^	1.826^ns^	1.536^ns^
*C*i (ppm)	0.274^ns^	0.196^ns^	0.507^ns^
*g*_s_ (mmol m^-2^s^-1^)	1.388^ns^	0.136^ns^	1.820^ns^
WUE (mmol mol^-1^)	4.228*	0.704^ns^	0.335^ns^
LN (mg g^-1^)	0.805 ^ns^	0.825 ^ns^	0.611 ^ns^
LP (mg g^-1^)	0.881 ^ns^	0.980 ^ns^	0.541 ^ns^
N/P	0.651 ^ns^	0.329 ^ns^	0.596 ^ns^
Chl (mg g^-1^)	1.028^ns^	5.476*	0.013^ns^
Chl *a/b*	3.892*	2.406^ns^	0.110^ns^
*F*_v_/*F*_m_	10.95^ns^	22.620**	0.638^ns^

*A*, net photosynthesis rate; *E*, transpiration rate; *C*i, intercellular CO_2_ concentration; *g*_s_, stomatal conductance; WUE, water use efficiency; LN, leaf nitrogen concentration; LP, leaf phosphorus concentration; N:P, leaf N:leaf P; and *F*_v_/*F*_m_, maximal quantum yield.

*, **, Significance levels:

*p≤0.05;

** p≤0.01;

^ns^ p>0.05, respectively.

**Table 3:** Growth parameters of *C*. *japonica* (Naidong) seedlings under different light intensity and nitrogen deposition treatments.

Parameters	L1			L2		
	N1	N2	N3	N1	N2	N3
Height (cm)	16.16±1.27ns	14.66±0.90ns	14.57±0.97ns	15.43±0.79ns	15.71±1.00ns	14.34±0.90ns
Basal diameter (cm)	0.45±0.03a	0.39±0.02b	0.36±0.02bc	0.37±0.02bc	0.35±0.02bc	0.32±0.01c
Crown area (cm^2^)	139.90±8.09a	144.6±9.59a	111.11±5.08b	93.64±5.67b	110.77±7.54b	96.85±7.37b

Values in the table are the mean ± SE of 8~9 replicates; different letters represent significant differences (*P*≤0.05) with Duncan’s multiple range test.

### Leaf traits

**Fig 1:**
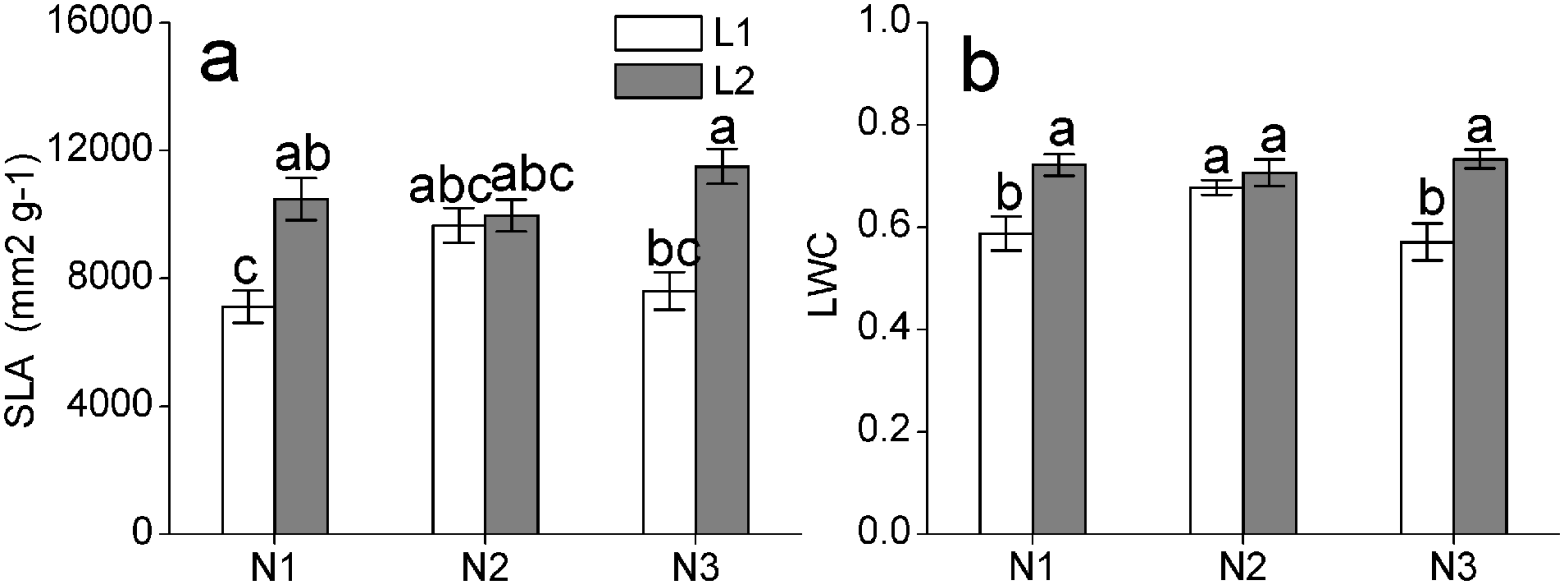
Leaf morphological traits of *C*. *japonica* (Naidong) seedlings under different light intensity and nitrogen deposition treatments. (a) Specific leaf area and (b) leaf water content. The values shown are the mean ± SE (*n* = 8–9). Different letters indicate a significant difference (p≤0.05) with Duncan’s multiple range test.

Specific leaf area and leaf water content showed markedly differences among light intensities and light × nitrogen deposition interaction as indicated by two-way ANOVAs (Table 2). For each nitrogen deposition rate, the specific leaf area and leaf water content under high irradiance were distinctly lower compared with those under low irradiance. However, no difference was observed under the moderate nitrogen deposition condition. Specific leaf area and leaf water content all increased first and then decreased with increasing of nitrogen addition in high light, but the tendency was opposite in low light (Fig 1a and 1b).

Leaf nitrogen and leaf phosphorus concentrations and leaf nitrogen:phosphorus ratio of seedlings were not significantly affected by light intensity, nitrogen deposition rate, and their interaction (Table 2).

### Photosynthetic pigment and chlorophyll fluorescence

**Fig 2:**
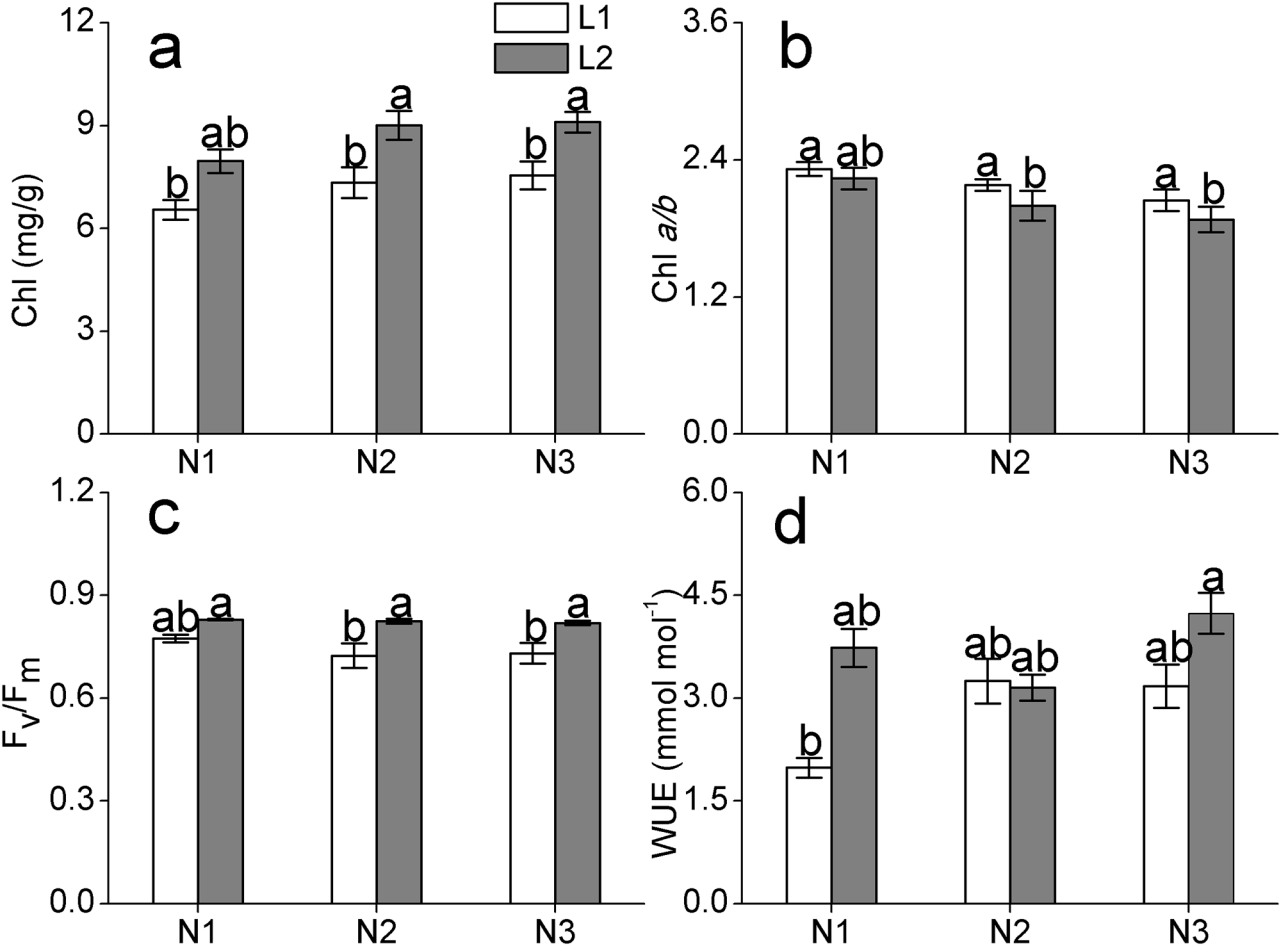
Photosynthetic pigment, chlorophyll fluorescence, and gas exchange parameters of *C*. *japonica* (Naidong) seedlings under different light intensity and nitrogen deposition treatments. (a) Chlorophyll content, (b) chlorophyll *a*/chlorophyll *b* ratio, (c) maximal quantum yield, and (d) water use efficiency. The values shown are the mean ± SE (*n* = 7–9). Different letters indicate a significant difference (p≤0.05) with Duncan’s multiple range test.

The irradiance intensity significantly affected chlorophyll content, but the chlorophyll *a/b* ratio was only significantly affected by nitrogen deposition (Table 2). The chlorophyll content was significantly higher in deep shade than in slight shade, and increased continuously with increasing rate of nitrogen deposition (Fig 2a). The chlorophyll *a/b* ratio was lower in deep shade compared with that in slight shade, which decreased with increase in the rate of nitrogen supply (Fig 2b).

Highly significant differences were observed in chlorophyll fluorescence under the different light intensities as indicated by two-way ANOVA (Table 2). The *F*_v_/*F*_m_ value was higher in deep shade than that in slight shade (Fig 2c).

### Gas exchange characteristics

No significant differences were observed for all gas exchange parameters, except water use efficiency (Table 2). Water use efficiency was influenced significantly by nitrogen addition rather than light intensity and their interaction. With increasing rate of nitrogen addition, water use efficiency increased consistently (Fig 2d).

## Discussion

In the present study, most parameters of Naidong seedlings were influenced by the shade or nitrogen deposition only. Water use efficiency and chlorophyll *a*/*b* of Naidong seedlings were response to light intensity significantly. In contrast, leaf water content, specific leaf area, chlorophyll content and maximal quantum yield were affected markedly by the nitrogen deposition. Only leaf water content was affected by the interaction of the shade and nitrogen deposition. Therefore, there were generally no significant interactions between light intensity and nitrogen supply rate on seedlings growth, leaf traits and physiological characteristics.

### Effects of light intensity

The influence of shade on plant growth can be broadly classified into two groups. Some studies hold that shade can limit plant growth owing to the lower relative growth rate and biomass accumulation [16, 35–38], whereas other studies indicate that there is a positive relationship between shade and plant growth [18, 21, 39]. In the present study, basal diameter and crown area were affected negatively by deep shade, which is in accordance with previous reports showing that low light intensity restricts seedlings growth and biomass accumulation [14, 16, 35, 40]. But what’s interesting about Naidong seedlings is that the height was not affected by the light intensity. This phenomenon contradicts that for *C*. *japonica* ‘Helen Bower’, whose height was, in fact, negatively influenced by deep shade [38].

Specific leaf area shows a negative relationship with leaf thickness; thinner leaves (leaves with a higher specific leaf area) require less photosynthetic machinery per unit area, hence show increased photosynthetic capacity [6, 41]. The specific leaf area is a good predictor for several physiological activities [14, 15, 42]. The specific leaf area was higher under the low irradiance condition than that under high irradiance, as reported in previous reports [14, 15, 35, 40, 42]. Higher specific leaf area may allow plants to better integrate light patches of different intensity in a heterogeneous light environment and therefore increase their total biomass [15, 42]. Changes in specific leaf area with light availability might be interpreted as a homeostatic mechanism to prioritize the optimization of light capture [14, 35, 40, 43].

Shade-acclimated leaves contain a higher concentration of chlorophyll per unit mass than sun-acclimated leaves. Leaves need to synthesize a greater quantity of chlorophyll to improve photosynthetic efficiency under low light [14, 18]. Previous studies have shown that shade can induce substantial changes in the contents of leaf photosynthetic pigments, of which total chlorophyll and the chlorophyll *a/b* ratio are important indicators for assessment of plant shade tolerance, and leaves of shade-tolerant plants show a high chlorophyll *a/b* ratio and low total chlorophyll content [16, 35]. In the present study, seedlings grown under low irradiance showed significantly increased chlorophyll content, indicating that plants show a shade-tolerant capability to some extent in order to maintain growth under low light environment [14]. As exhibited in previous studies, the marked increase in leaf chlorophyll content in deep shade demonstrates a plant’s ability to maximize the light-harvesting capacity under low-light growth conditions [35, 38, 44, 45]. In addition, the higher chlorophyll *a/b* of Naidong seedlings under the high light level indicated that the greater investment in chlorophyll b improve PSII function in the shading [14, 35].

The maximal PSII quantum yield (*F*_v_/*F*_m_) is the primary target of photoinhibition. It is considered to be a reliable indicator of PSII activity, which is usually stable for a healthy leaf [46–49]. The *F*_v_/*F*_m_ value close to 0.8 is considered to be a reference value for healthy leaves [6, 50]. In non-stressful environmental conditions, the fraction of light energy used for photochemistry decreases and the fraction of light energy used for fluorescence increases with increasing light intensity [49, 51, 52]. In the present study, the *F*_v_/*F*_m_ values fluctuated around 0.8 under low illumination conditions, but declined significantly under high illumination conditions. This result may be because the physiological state of PSII and the photosynthetic organs of the seedlings were damaged in a high irradiance environment. The present results illustrated that low light intensity enhanced the adaptability of photosynthetic components, and the electronic plant photochemical efficiency and light reaction transfer efficiency were also improved.

The present-day light environments on the islands that host natural Naidong populations have been substantially modified due to human activities, such as tourism and exploitation of the natural resources of these islands. Moreover, Naidong plants are exposed to increasingly heterogeneous light environments as the companion species are disappearing. Once exposed to the intense light irradiation, the growth of Naidong seedlings would be inhibited because the seedlings leaves will be scorched [27]. Therefore, development of a management plan or conservation policy for Naidong should not be undertaken without consideration of its companion species. The protection of associated species to improve biodiversity will provide ideal light environments for Naidong seedlings.

### Effects of nitrogen deposition

In the present study, the nitrogen deposition had relatively little impact on seedling growth, which is in contrast to the results of previous researches [6, 11, 12]. The reason may be explored combined with variation in other leaf trait parameters (net photosynthetic rate, leaf nitrogen concentration, and chlorophyll content) under the same nitrogen addition treatments. A previous study reported that 75% of the nitrogen content of the leaves of a C_3_ plant is used in the chloroplasts, of which the majority was used in photosynthesis [53]. Leaf nitrogen concentration and chlorophyll content directly affect the photosynthetic rate. The leaf nitrogen concentration and chlorophyll content were not affected by nitrogen supply in the current study, which is in contrast to previous findings [3, 6, 11]. Therefore, we speculated that the chlorophyll content and leaf nitrogen concentration were unaffected by nitrogen load, which may explain why the net photosynthetic rate was unchanged with increasing nitrogen availability. Moreover, another reason is the slow growth rate of Naidong seedlings.

The phenomenon by which the crown area of seedlings initially increased and subsequently declined with increasing rate of nitrogen addition indicated that there is a threshold for the effect of nitrogen on Naidong seedlings. The present results are in agreement with the conclusion that the heights of *Quercus acutissima*, *Q*. *variabilis* and *Q*. *mongolica* seedlings were unaffected by nitrogen deposition [11, 12, 14], but conflict with certain previous studies that the nitrogen supply significant affects the height of *Ailanthus altissima* and *Acer truncatum* seedlings [6, 18]. We speculated that the reason may be the slow growth of Naidong. According to our results, Naidong seedlings were insensitive to short-term nitrogen deposition, therefore, it is necessary to carry out a long-term test to verify that.

Water use efficiency, defined as the ratio of photosynthetic carbon assimilation over transpiration, is widely recognized to be a critical link between carbon and water cycling in terrestrial ecosystems [54]. Although some physiological parameters did not differ significantly under the three nitrogen deposition treatments, higher water use efficiency was observed under high nitrogen deposition, which is in accordance with previous findings [3]. Increasing the concentration of a variety of salt ions in the soil results in osmotic stress, and thus plants suffer resistance to water absorption. This may be a reason why water use efficiency increases with increasing nitrogen availability [2, 3, 55]. Increasing nitrogen supply improves water use efficiency, which is beneficial to maximize utilization of resources in the case of sufficient resource availability, or maintain essential physiological functions under conditions of limited resource availability.

Although the growth of Naidong seedlings in natural environments depends on many ecological factors, enhanced nitrogen deposition has negative effects on growth in Naidong populations in the Yellow Sea of China. In the long term, Naidong seedlings will probably be more severely affected by future nitrogen deposition, which should be tested in additional experiments.

### Combined effects of shade and nitrogen deposition

In the present study no significant interactions between shade and nitrogen supply on plant growth and the physiological parameters were observed, which is in accordance with researches on *Deschampsia flexuosaas* and *Q*. *acutissima* [14, 21]. Previous studies have reported that the growth, photosynthetic characteristics, and quality of plants are affected by interactions between light intensity and nitrogen availability [15, 18, 20, 56]. Nitrogen addition increases the dry biomass of lettuce under low irradiance, but decreases dry biomass under high irradiance [18]. The reasons for these results were that the leaf photosynthetic rate of lettuce is elevated with increasing irradiance, and excessive nitrogen supply results in adverse effects on plant photosynthesis because of nutrient imbalance [18]. Nitrogen supply increases the height and total dry weight of *Pinus pinaster* under high and medium light intensities, but nevertheless did not affect those indices under low light intensity [56]. In contrast, high irradiance and high nitrogen supply enhance the growth of five tropical dry forest tree species compared with other treatments [19]. However, a consistent conclusion on the interaction of light and nitrogen deposition on plants is still lacking. In addition, an individualistic and differential response by each species was apparent, so a single species study is not strong enough to achieve a general conclusion.

We consider that the leaf water content of Naidong is more sensitive to various light intensity and nitrogen deposition treatments compared with other parameters. The reason for this may be that excess or deficient nitrogen can lead to leaf ion imbalance [6, 11], resulting in changes to leaf water potential, and that light intensity affects the leaf transpiration rate [6, 40], resulting in changes to the leaf water content.

We speculate that the mechanisms by which Naidong responds to light intensity and nitrogen addition treatments may be totally different. Generally, no response to the interaction of light intensity and nitrogen deposition by Naidong seedlings was observed.

## Conclusions

In summary, the present study showed that nitrogen deposition had not alleviated the adverse effects of shade in Naidong seedlings. Slight shade is helpful to the growth of Naidong, and Naidong seedlings can acclimate to deep shade by increasing the chlorophyll content, leaf water content and specific leaf area. The increasing of chlorophyll content of Naidong leaves enhanced the capacity of capture light under deep light condition. Moreover, the higher leaf water content and specific leaf area at low light environments indicated that Naidong seedlings can invest more resources into photosynthesis and growth. Moderate nitrogen supply can promote growth and improve the water use efficiency of Naidong seedlings. This study also indicated that nitrogen deposition had no effect on physiological parameters of seedlings due to their slow growth and the brief duration of this experiment. Nevertheless, the growth of the seedlings was inhibited by excess nitrogen addition, and we believe that there is a threshold for the effects of nitrogen load on Naidong seedlings growth. Moderate nitrogen addition could slightly alleviate the negative effects of drought on plants under the moderate or severe drought conditions. In the cultivation management, we therefore recommend the accumulation of nitrogen in the soil should be controlled when the soil moisture is sufficient. A proper amount of nitrogen can be added to improve the drought resistance of Naidong seedlings under the water deficit conditions, and nitrogen addition can alleviate the negative effects of the drought on seedlings. Last but not least, establishment of nature reserves is needed to protect the natural habitats of Naidong and its companion species, and extant Naidong populations should be protected by means of ex situ conservation and collection of germplasm resources to preserve genetic diversity of Naidong outside of established nature reserves.

## Supporting information

S1 Table Relevant data underlying the findings described in manuscript. (DOCX)
