# Supplementary material for: Nitrogen deposition does not alleviate the adverse effects of shade on Camellia japonica (Naidong) seedlings
Source: PLoS One. 2018 Aug 9;13(8):e0201896. doi: 10.1371/journal.pone.0201896 (PMC6084955; doi:10.1371/journal.pone.0201896)
Supplement: S1 Table — (DOCX) [file pone.0201896.s001.docx]

**S1 Table. Relevant data underlying the findings described in manuscript.**

| Parameter |  | L1 |  |  | L2 |  |
| --- | --- | --- | --- | --- | --- | --- |
|  | W1 | W2 | W3 | W1 | W2 | W3 |
| *A* (μmol m^-2^s^-1^) | 1.26±0.34 | 2.44±0.49 | 1.54±0.26 | 2.06±0.65 | 1.73±0.43 | 2.00±0.61 |
| *E* (mmol m^-2^s^-1^) | 0.51±0.07 | 0.71±0.09 | 0.45±0.05 | 0.47±0.06 | 0.48±0.08 | 0.47±0.08 |
| *C*_i_  (ppm) | 296.75±18.78 | 270.89±20.70 | 261.857±14.83 | 280.00±24.97 | 289.43±17.74 | 283.00±27.96 |
| *g*_s_ (mmol m^-2^s^-1^) | 17.63±2.59 | 29.00±4.12 | 19.43±2.08 | 23.86±3.73 | 22.71±3.93 | 22.67±4.33 |
| LN  (mg g^-1^) | 18.32±4.86 | 13.45±5.23 | 17.17±3.71 | 17.90±2.29 | 18.44±3.92 | 14.95±1.98 |
| LP  (mg g^-1^) | 10.16±1.25 | 8.13±1.13 | 9.10±2.45 | 8.05±0.54 | 10.86±3.20 | 9.25±2.34 |
| N/P | 2.12±0.85 | 1.43±0.43 | 2.55±0.38 | 2.25±0.44 | 1.50±0.55 | 1.77±0.31 |

*A*, net photosynthesis rate; *E*, transpiration rate; *C*i, intercellular CO_2_ concentration; *g*_s_, stomatal conductance; LN, leaf nitrogen concentration; LP, and leaf phosphorus concentration; N:P, leaf N:leaf P. The values shown are the mean ± SE.
